# Group A* Streptococcus* Septic Shock after Surgical Abortion: A Case Report and Review of the Literature

**DOI:** 10.1155/2017/6316739

**Published:** 2017-09-11

**Authors:** Stephanie C. Tardieu, Elizabeth Schmidt

**Affiliations:** Department of Obstetrics and Gynecology, Hofstra Northwell School of Medicine, Hofstra University, North Shore-LIJ University Hospital, 270-05 76th Avenue, New Hyde Park, NY 11040, USA

## Abstract

Group A* Streptococcus* (GAS) causing puerperal sepsis is a leading cause of maternal mortality worldwide. Although rare, GAS infection is a relatively significant public health concern because of its propensity to evolve rapidly into septic shock, streptococcal toxic shock syndrome, and death. We report the case of a 27-year-old patient who presented with GAS septic shock after undergoing a surgical termination of pregnancy and was treated successfully and recovered without sequelae. GAS septic shock should always be included in the differential diagnosis of any patient who develops sepsis after a surgical abortion. Patients with GAS septic shock have a rapid clinical decline and need aggressive fluid management, early initiation of broad-spectrum antibiotics, and rapid surgical intervention.

## 1. Introduction

Group A* Streptococcus* (GAS) infection is a leading cause of death from puerperal sepsis worldwide [1]. Prior to the antibiotic era, GAS infections were a major cause of peripartum morbidity and mortality [2]. Currently, approximately 246 cases of puerperal GAS infection are reported every year in the United States [3].

Although its incidence remains relatively rare, GAS infection is significant because of its potential to progress rapidly to septic shock, organ damage, and streptococcal toxic shock syndrome (TSS), which are associated with significant morbidity and mortality. Patients with invasive GAS infections who develop septic shock, streptococcal TSS, or necrotizing fasciitis have mortality rates of 45%, 38%, and 29%, respectively [3].

This is the first case report of invasive GAS infection after a surgical abortion. We present the clinical presentation, management, and treatment of a patient who presented with septic shock secondary to GAS infection after a first-trimester surgical termination of pregnancy.

## 2. Case Report

A 27-year-old gravida 6, para 3033 female was brought to the emergency room with a fever and severe lower abdominal pain that had begun 5 hours earlier. She had undergone a dilation and curettage at 6 weeks' gestation 24 hours prior to presentation at a private outpatient family planning clinic unaffiliated to our institution. Her pain was sharp and diffuse and associated with nausea, vomiting, rigors, and dyspnea. She denied rashes, vaginal bleeding, or abnormal vaginal discharge.

Upon presentation to the emergency room, the patient's vital signs were as follows: blood pressure of 130/69, heart rate of 131 beats per minute, respiratory rate of 18 breaths per minute, oxygen saturation on room air of 100%, and temperature of 38.0°C (100.4°F). Within the hour, she became hypotensive with a blood pressure of 77/54, heart rate of 111, respiratory rate of 20, and oxygen saturation of 99% on room air. Her temperature rose to 39.6°C (103.4°F), and her blood pressure dropped to 60/30 during resuscitative efforts.

The patient appeared pale and diaphoretic but was alert, awake, and oriented. Lung fields were bilaterally clear without rales, wheezes, or rhonchi. Her abdomen was soft and nondistended with diffuse tenderness to light palpation and positive rebound and guarding. Pelvic examination revealed an open cervix with copious amounts of opaque mucous discharge draining from the cervical os with no foul odor. The uterus was 6 weeks in size, and there were severe cervical motion and uterine tenderness on bimanual examination.

Initial labs were as follows: white blood cell count (WBC), 9.4 × 10^3^/mm^3^; hemoglobin, 11.9 g/dL; hematocrit, 34.1%; platelets, 257 × 10^3^/mm^3^; and lactate, 2.5 mg/dL. Creatinine, liver function tests, and coagulation profile were normal. Transvaginal ultrasound (TVUS) revealed a normal-sized uterus with the endometrial lining measuring 9.5 mm containing heterogeneous material that was negative to Doppler interrogation. A computed tomography (CT) scan of the abdomen and pelvis with contrast was negative for intra-abdominal pathology.

Her past medical history included asthma and rheumatoid arthritis, and she had a penicillin allergy producing angioedema.

In the emergency room, two intravenous lines (IVs) were started, and the patient was resuscitated with three liters of Lactated Ringer. She was started on IV ertapenem and metronidazole for presumed endometritis. A Foley catheter was inserted. Given her continued hemodynamic instability despite adequate fluid resuscitation, the decision was made to perform an emergency dilation and curettage and diagnostic laparoscopy.

Intraoperatively, no gross abnormalities of the uterus, adnexa, bowel, appendix, gall bladder, or liver were seen. Dilation and curettage produced minimal hemorrhagic tissue that was sent to pathology. The patient remained hypotensive and was treated with dopamine for vasopressor support. The intraoperative lactate level was 4.5 mg/dL.

Postoperatively, the patient was admitted to the surgical intensive care unit. Her lactate level postoperatively was 4.6 mg/dL. On postoperative day 1, she continued to report severe, diffuse abdominal pain with rebound and guarding. She was febrile with a maximum temperature of 38.8°C (102°F) and continued to require dopamine for vasopressor support. Metronidazole was discontinued and vancomycin was started. Her lactate level decreased to 1.1 mg/dL, and her WBC was 10.9 × 10^3^/mm^3^.

On postoperative day 2, the patient remained febrile with a temperature of 39.1°C (102.5°F) with decreased abdominal pain. She was weaned off vasopressor support. Her cervical cultures and blood cultures from admission were positive for group A beta-hemolytic* Streptococcus* from both tubes. Antibiotics were changed from vancomycin to clindamycin. Her WBC dropped to 4.2 × 10^3^/mm^3^.

On postoperative day 3, the patient's abdominal pain continued to improve. She had fundal tenderness without rebound or guarding. She was afebrile for 24 hours and was transferred to the inpatient gynecology floor. A second set of blood cultures drawn on postoperative day 2 were negative for GAS. She remained afebrile on postoperative day 4 and was discharged on postoperative day 5 with a peripherally inserted central catheter line and IV ertapenem to be continued for 14 days. No retained products of conception were found on pathology.

The patient provided written informed consent for the treatment and for publication of this case.

## 3. Discussion

Surgical abortion is a safe and reliable surgical procedure for termination of pregnancy. The overall frequency of infections following surgical abortion in the first trimester is 0.27% [4]. Case reports of invasive GAS infection in the literature include infection in the puerperal period as well as after medical abortion [5, 6]. US Centers for Disease Control data from 2005 to 2012 estimates that the incidence of puerperal invasive GAS infection is approximately 246 cases per year with a mortality rate of 10.6% [3]. However, to our knowledge, this is the first case report being published on GAS sepsis after a surgical abortion.

Puerperal GAS infections vary in presentation from mild endometritis to invasive life-threatening infections [7]. The classic presentation of puerperal GAS infection is a woman who presents within 48 hours from delivery or abortion with fever, rigor, and severe abdominal pain. Clinical findings can include purulent vaginal discharge, uterine tenderness, nausea, and vomiting [7]. A key characteristic that distinguishes GAS sepsis from other types of sepsis is the patient's rapid clinical deterioration despite appropriate resuscitative measures with fluids and broad-spectrum antibiotics [8]. The presence of hypotension, tachycardia, and leukocytosis are signs that TSS is developing, which is associated with higher mortality rates [3, 9].

Toxic shock syndrome (TSS) develops in up to 14–20% of patients with invasive GAS infection [10]. Its diagnosis carries a poor prognosis and a high mortality rate [3, 9]. Diagnosis of TSS is made when GAS is isolated from a sterile (blood, cerebrospinal fluid, pleura, peritoneal fluid, tissue biopsy, or a surgical wound) or nonsterile site (vagina, throat, sputum, or skin) in a patient with hypotension (systolic blood pressure < 90) who has at least two of the following organ dysfunctions: renal impairment (creatinine > 2 mg/Dl), coagulopathy (platelets < 100,000 mm^3^ or disseminated intravascular coagulopathy), liver impairment (LFTs > 2x the upper limit of normal), acute respiratory distress syndrome, generalized erythematous macular rash that may desquamate, and/or soft tissue necrosis [11].

In puerperal or postabortion GAS sepsis, necrosis of the uterus and adnexa may be present [12]. Atypical presentations include necrotizing fasciitis involving the limbs and cervix [6, 12, 13]. Cases of puerperal TSS causing myositis, rhabdomyolysis, disseminated intravascular coagulopathy, and acute respiratory distress syndrome have been reported [14]. Rare complications may include septic ovarian thrombosis and septic arthritis [15]. Early diagnosis of complications associated with invasive GAS infection is vital, as early surgical intervention by debridement or hysterectomy may be necessary. Surgical intervention with hysterectomy is crucial when signs of deeper invasion are present and necrosis is suspected [16].

Clinicians should have a high index of suspicion for invasive GAS infection when evaluating a patient with postabortion sepsis. Work-up should include a complete blood count with differential, renal, and liver function tests, coagulation profile (PT/INR, aPTT, and fibrinogen), lactate, and cervical and/or wound cultures at presentation (Table 1) [7]. A swab or blood culture positive for GAS is necessary for diagnosis but should not delay aggressive management with fluid resuscitation and broad-spectrum IV antibiotics [17]. Gram stain will show Gram-positive cocci in chains or in pairs. Bandemia > 10% may be present even in the absence of leukocytosis [1]. Hemoconcentration or hemolysis may also be present [1]. Arterial blood gases are important for monitoring lactate, pH, and arterial oxygenation levels. A chest X-ray should be ordered for patients with dyspnea and low oxygen saturation. Other sources of infection should also be ruled out including urinary tract infection, pyelonephritis, and endometritis. Imaging with CT, magnetic resonance imaging, or TVUS provides clinical information about the presence of an abscess and retained products of conception. However, normal imaging should not delay aggressive management.

Imaging may demonstrate an edematous uterus that is larger than expected. GAS does not usually create abscesses or produce gas. The presence of gas in the endomyometrium may point to another bacterial etiology for postabortion sepsis or septic shock such as infection with* Clostridium sordellii*. These infections typically lack a fever and produce a leukemoid reaction (WBC 50–200 × 10^3^/mm^3^) and diffuse capillary leakage [18].

Early treatment of GAS sepsis requires antibiotic therapy combined with aggressive fluid resuscitation. Once shock develops, the mortality rate approaches 45–60% [3, 9]. Infectious disease consultation should be obtained as soon as possible. IV benzylpenicillin (4 million units every 4 hours) is the first-line antibiotic. Clindamycin should be added in cases of severe sepsis and septic shock (900 mg every 8 hours). Patients with a penicillin allergy, as was the case for this patient, can be treated with a broad-spectrum cephalosporin and vancomycin (15 mg/kg every 12 hours) [19]. The length of treatment should be individualized and determined in collaboration with infectious disease specialists. Patients with positive blood cultures should be treated for at least 14 days.

Given the critical nature of invasive GAS infections, rapidly deteriorating women require treatment in an intensive care unit [20]. When TSS is present, volume deficits are large and can require up to 10 to 20 liters of fluid resuscitation per day [21]. Vasopressor therapy will usually be required. Strict monitoring of fluid intake and output is important to prevent sequelae of fluid overload. If the patient continues to deteriorate despite antibiotic therapy and adequate fluid resuscitation, it is necessary to consider deeper signs of infection such as necrosis. Surgical intervention is required when a confirmed GAS infection is present with signs of organ dysfunction [9]. In our case, diagnostic laparoscopy and dilation and curettage were performed. Necrosis and gangrene were not observed in our patient; however, there are cases of necrosis and necrotizing fasciitis of the uterus and adnexa requiring hysterectomy, adnexectomy, and debridement of necrosed tissues [12, 22]. The use of intravenous immunoglobulin in the treatment of puerperal GAS sepsis is debated; however, most favor its administration in patients with TSS to boost the patient's passive immunity [23, 24].

Given its rarity, screening of carriage for GAS is not thought to be helpful in the prevention of invasive disease. Thus far, optimal prevention and vaccination are not yet available. GAS transmission can occur from inhalation or direct contact with droplets of saliva and nasal or vaginal secretions. It can also occur through skin-to-skin contact with those who have infected lesions. The need for rigorous personal hygiene and hand washing should be emphasized to the patient, her close contacts, and healthcare workers who are caring for her. Although rare, healthcare workers can acquire GAS infections from patients. The CDC has issued guidelines for screening and prophylaxis to prevent transmission of invasive GAS infections to household contacts and to persons involved in their healthcare [8, 25].

Healthcare workers should always wear protective equipment including gloves, masks, and gown when directly caring for the patient [8]. Patients should be placed in an isolated room with contact precautions and strict hand hygiene practices should be employed. Close contacts and healthcare workers who have a sore throat, skin infection, vaginitis, or a needle stick within the 30-day period spent treating the patient should receive screening and an evaluation by an infectious disease specialist [8]. Discussion about who requires screening and antibiotic regimens for a positive screen are beyond the scope of this paper but can be found in our referenced literature [25].

## 4. Conclusion

This presentation of a patient who developed postabortion GAS septic shock highlights the fact that these patients can deteriorate rapidly and require aggressive fluid management, early initiation of broad-spectrum antibiotics, and concurrent rapid surgical intervention. Although the patient in this report did not develop streptococcal TSS, she did develop septic shock with a rapid clinical decline. We believe that she would have developed TSS had she not received timely aggressive medical and surgical intervention. Early involvement of infectious disease, critical care, and surgical specialists were vital to the patient's positive outcome, highlighting the need for an interdisciplinary approach when caring for patients with GAS sepsis. GAS infection should be included in the differential diagnosis of postabortion sepsis, as it requires aggressive management and an interdisciplinary approach to avoid life-threatening septic shock, TSS, end-organ failure, and death.

## Figures and Tables

**Table 1:** Diagnostic work-up for postabortion sepsis.

Blood work	CBC w/differential
	CMP
	Coagulation profile (PT/INR, aPTT, fibrinogen)
	Lactate
	Arterial blood gas

Imaging	Chest X-ray
	Transvaginal ultrasound
	CT abdomen and pelvis with contrast or MRI

Cultures	Blood cultures
	Cervical cultures
	Vaginal discharge cultures (including cultures for trichomonas, gonorrhea, chlamydia, and candida)
	Cultures of endometrial biopsy or curettage specimen
	Urine culture

Other tests	Urinalysis

Consultation	Gynecology (including family planning subspecialist if available)
	Infectious disease
	Hospital infectious control services
	Surgery (general and other subspecialists)
	Critical care

CBC: complete blood count; CMP: comprehensive metabolic panel; PT/INR: prothrombin time/international normalized ratio; aPTT: activated partial thromboplastin time; CT: computed tomography; MRI: magnetic resonance imaging.
